# Effects of Regulated Deficit Irrigation on Amino Acid Profiles and Their Derived Volatile Compounds in Cabernet Sauvignon (*Vitis vinifera* L.) Grapes and Wines

**DOI:** 10.3390/molecules23081983

**Published:** 2018-08-09

**Authors:** Yan-lun Ju, Guo-qian Xu, Xiao-feng Yue, Xian-fang Zhao, Ting-yao Tu, Jun-xiang Zhang, Yu-lin Fang

**Affiliations:** 1 College of Enology, Northwest A & F University, Yangling 712100, China; juyanlun2016@nwsuaf.edu.cn (Y.-l.J.); yuexiaofeng@nwsuaf.edu.cn (X.-f.Y.); zhaoxianfang@nwsuaf.edu.cn (X.-f.Z.); tuty@lzlj.com (T.-y.T.); 2 Ningxia Grape and Wine Research Institute, Ningxia University, Yinchuan 750000, China; xugql@163.com; 3 Shaanxi Engineering Research Center for Viti-Viniculture, Yangling 712100, China

**Keywords:** water deficit irrigation, secondary metabolism, red wine, winegrape

## Abstract

Amino acid contents and their derived volatile compositions in Cabernet Sauvignon grapes and wines after regulated deficit irrigation (RDI) were investigated during the 2015 and 2016 growing seasons in Yinchuan (NingXia, China). High-performance liquid chromatography (HPLC) and gas chromatography-mass spectrometry (GC-MS) were used for amino acid and volatile compound analyses. Three RDI strategies were tested: 60% (RDI-1), 70% (RDI-2), and 80% (RDI-3) of grapevine estimated evapotranspiration (ETc), and 100% ETc was used as the control group (CK). RDI-treated vines had lower yields and berry weights with higher total soluble solids than the control treatment. RDI-1 increased proline levels in berries and wines. RDI-2 enhanced tyrosine and asparagine levels in wines. RDI-3 enhanced arginine, alanine, valine, leucine, and isoleucine levels in berries and wines. RDI-2 and RDI-3 increased the concentrations of 2-methyl-1-butyl acetate, benzaldehyde, 3-methyl-1-pentanol, and 3-methyl-1-butanol in wines. The accumulation of volatile compounds was closely related to the amino acid concentrations—especially isoleucine, valine, and leucine—in grapes. Our results showed that RDI treatments altered amino acid concentrations and their derived volatile compositions in wines.

## 1. Introduction

Amino acids present in grapes serve as a nitrogen source for yeasts during alcoholic fermentation [1]. Amino acids in grapes are precursors of volatile compounds, such as leucine, valine, isoleucine, and phenylalanine [2]; and the branched-chain amino acids (valine, leucine, and isoleucine), aromatic amino acids (phenylalanine, tyrosine, and tryptophan), and methionine in grapes can be converted into *α*-ketoacids and metabolized into higher alcohols and higher acids in yeast cells through the Ehrlich pathway [2,3]. These compounds can be further metabolized into alcohol esters and acetates [3]. These fermentative volatile compounds account for approximately 50% of the total volatile concentration in wines [4]. Thus, amino acid profiles in grapes affect fermentation development and have an important role in the production of volatile compositions [2]. Many previous studies have reported how changes in grape amino acid content can affect wine volatile composition [1,5,6] because phenylalanine, leucine, isoleucine, valine, tyrosine, and methionine seem to be precursors of volatile compounds (2-phenylethanol, 3-methyl-1-butanol, 2-methyl-1-butanol, isobutanol, tyrosol, and methionol, respectively). The addition of branched-chain amino acids (such as valine, leucine, and isoleucine) during fermentation increases higher alcohol production. In comparison, adding phenylalanine increases the concentrations of 2-phenylethanol, 2-phenylethyl acetate, and ethyl esters in wine [7]. Therefore, future work is needed to reveal the relationship between amino acids and aromas in grapes and wines. Amino acids and volatile compositions in grapes and wines are affected by many factors, including viticultural practices, climate conditions, and nitrogen fertilization [7,8,9,10]. Recently, great research effort has been devoted to assessing changes in the amino acids and aroma components in grapes and wines after different treatments. Sánchez-Gómez et al. [8] reported that the application of aqueous extracts of vine-shoot waste could enhance the amino acid content of wine. They also reported that a higher amino acid concentration in must resulted in higher consumption during alcoholic fermentation, and the resultant wines have a lower concentration of amino acids. Bouzas-Cid et al. [9] reported that irrigation alters the concentration of methionine in musts, and the vintage is closely related to the amino acid content in must.

Grape is an important fruit with economic and nutritional value and is cultivated in China. Northwest China is the main grape cultivation area, especially Ningxia, Xinjiang, and Gansu. However, the shortage of fresh water resources restricts the development of the grape industry in China. Regulated deficit irrigation (RDI) can save water and improve crop quality [11,12,13], alter grape and wine flavors and phenolic compounds, and plays an important role in improving vine canopy shapes, vigorous vines, and canopy microclimates [14,15,16,17,18]. However, few reports exist on the effect of RDI on amino acid contents and their derived volatiles in grapes and wines. Exploring whether this influence is positive or negative could provide a theoretical foundation for producing high-quality grapes and wines.

Consequently, the aim of this work was to gain insight on the effect of RDI grapevine treatment on the amino acid composition in grapes and wine and fermentative volatile compounds in wine since previous studies suggest that using RDI as a strategy to promote efficient water use in agriculture alters metabolites in grapes. The relationship between amino acids and the fermentative volatile compositions in wine was also elucidated using three different RDI strategies.

## 2. Materials and Methods

### 2.1. Field Conditions and Materials

The study was conducted with own-rooted red wine grape (*Vitis vinifera* L.) cv. Cabernet Sauvignon from a commercial vineyard (Chateau Lilan, 38°28′ N 105°97′ E, elevation 1169 m, semiarid continental climate) in Yinchuan (Ningxia, China). The experiments were conducted for two continuous vintages from 2015 to 2016. Vines were oriented in the north–south direction with drip irrigation (2.4 L/h, irrigation time determined by the amount of irrigation). The vines were spaced at 3.0 × 0.6 m and had been planted in 2010. A completely randomized block design using three replicates of five lines with 100 vines each (Three central lines were used for experiments and sampling, whereas the remaining lines acted as buffers.) was performed. Three RDI strategies were used: 60% (RDI-1), 70% (RDI-2), and 80% (RDI-3) of the estimated evapotranspiration (ETc), and 100% ETc was used for the control group (CK). The vines were irrigated from fruit set until approximately two weeks prior to harvest. Irrigation was applied when vines reached a midday stem water potential value of −0.6 MPa. The ETc was calculated using the Pennman–Monteith model [19], and meteorological data were obtained from a microweather station located within the experiment vineyard. Vine water status was monitored by measuring the leaf water potential at midday (ψmd) using a pressure chamber after treatments.

### 2.2. Samples and Vinifications

When the grapes attained 22–24 Brix, samples were manually collected for each replicate on the same day and transported to the laboratory in ice boxes within 30 min. In total, 600 grapes were collected for each replicate. The yield was measured, and one hundred grapes were used to analyze the physicochemical parameters according to International Organisation of Vine and Wine [20] methods. Samples were immediately frozen in liquid nitrogen and stored at −40 °C before further analysis. All treatments were carried out in triplicate, and the results are the average of three analyses (*n* = 3).

Approximately 100 kg of grapes from each treatment group were used for vinification in stainless steel tanks, and all vinifications were performed in triplicate. Before alcoholic fermentation (AF), the total acidity of must was adjusted to 5.5 g/L, and pectolytic enzymes (30 mg/L) and SO_2_ (50 mg/L) were added. After 24 h, a commercial yeast *Saccharomyces cerevisiae* strain BO 213 (200 mg/L, Laffort, Bordeaux, France) was added according to direction. All the fermentation was controlled at the same condition (25 ± 1 °C). The temperature and must density were automatically monitored. At the end of alcoholic fermentation and without malolactic fermentation, filtration, tartaric stabilization, and natural clarification were manually performed, and the resultant wine samples were collected and stored at −40 °C before further analysis.

### 2.3. HPLC Determination of Amino Acids

#### Derivatization of Amino Acids

The HPLC analysis was performed using a Shimadzu LA-20AT system (Shimadzu, Kyoto, Japan) equipped with an automatic liquid sampler. Chromatographic separation was performed on a C18 column (AJS-01, 3 μm, 150 × 4.6 mm; Agela Technologies, Tianjin, China). The column was operated at 30 °C. The derivatization of amino acids was carried out according to the methods of Sánchez-Gómez et al. [8] and Liu et al. [21]. Briefly, fifty grape berries were deseeded and turned into powder under liquid nitrogen. Grape musts were centrifuged at 4 °C and 10,000 rpm for 10 min to collect clear juice. For precolumn derivatization, the clear juice or wine was filtered through 0.22 μm nylon 66 organic membranes (Micro Pes, Membrana, Wuppertal, Germany), and 100 μL of norvaline and 100 μL of sarcosine (internal standards) were added to each sample (5 mL). The samples were then submitted to an automatic precolumn derivatization with o-phthaldialdehyde (OPA reagent 1, Agilent) for primary amino acids and with 9-fluorenylmethylchloroformate (FMOC reagent 2, Agilent) for proline. 10 μL from the derivatized sample were injected at 40 °C, and total reaction time was about 90 s.

Two eluents were used as the mobile phases: eluent A: 75 mM sodium acetate, 0.018% triethylamine (pH 6.9), and 0.3% tetrahydrofuran; eluent B: ultra-pure water, methanol, and acetonitrile (10:40:50 *v/v/v*). Phases A and B were filtered through 0.22 μm nylon-66 organic membranes.

All samples were eluted according to a previous method reported by Garde-Cerdán, et al. [22] with some modifications. The total analysis time was approximately 40 min. Briefly, a 1.6 mL/min flow rate was used with the following elution program: 6% B for 10 min, 6 to 10% B in 6 min, maintained for 2 min, from 10 to 16% B in 2 min, from 16 to 40% B in 13 min, from 40 to 50% B in 7 min, from 50 to 100% B in 1 min, and maintained for 4 min. The elution program was followed by balancing the column with Phases A and B for about 1 min. The injection amount was 10 μL, and for detection, a photodiode array detector monitored for primary and secondary amino acids at 338 and 262 nm, respectively. The identification and quantification of amino acids were performed using pure reference standards. All external chemical standards (tryptophan (trp, 98%), glycine (gly, 98.5%), phenylalanine (phe, 98%), methionine (met, 98%), leucine (leu, 98%), arginine (arg, 98%), proline (pro, 99%), lysine (lys, 98%), glutamic acid (glu, 99%), asparagine (asp, 99%), isoleucine (ile, 99%), valine (val, 98%), histidine (his, 98%), serine (ser, 98%), alanine (ala, 98%), threonine (thr, 98%), and cysteine (cys, 97%)) used for the identification and quantification of amino acids were purchased from Sigma-Aldrich (Shanghai, China). The concentrations of the amino acids of the standard are shown in Table S1.

### 2.4. Determination of Volatile Compounds by GC-MS

#### 2.4.1. Extraction of Volatile Compounds

The extraction of volatile compounds was carried out according to previous research [23]. Briefly, 100 randomly selected grapes were deseeded and ground under liquid nitrogen. Fifty grams of the obtained powder was mixed with 1 g polyvinylpolypyrrolidone (PVPP), and the mixture was macerated at 4 °C for 3 h under a nitrogen atmosphere. Then, the mixture was centrifuged at 8000 rpm (4 °C) for 20 min. The clear juice or wine was filtered twice through 0.45 μm nylon 66 organic membranes. For volatile compound determination, 10 mL of wine with 40 μL of 2-octanol as an internal standard (0.32 g/L dissolved in ethanol, Sigma-Aldrich (Shanghai, China) was blended with 2 g NaCl in a 20 mL sample vial containing a magnetic stirrer. The vial was tightly capped with a PTFE-silicon septum for further analysis.

#### 2.4.2. GC-MS Analysis

GC-MS analyses were performed using a Thermo-Finnigan Trace 2000 gas chromatograph (Thermo Finnigan, Waltham, MA, USA) with an HP-INNWAX column (60 m, 0.25 mm I.D., 0.25 μm; Agilent, Shanghai, China). The solid-phase micro-extraction fiber (SPME, DVB/CAR/PDMS 2CM, 50/30-μm) was heated at 250 °C for 2 h. The vial containing the sample (wine) was extracted in a 40 °C water bath for 30 min while being stirred and then desorbed at 230 °C for 3 min into the splitless injection port of the GC-MS instrument. Helium was used as the carrier gas (1 mL/min). The chromatographic conditions consisted of an initial oven temperature of 40 °C for 2.5 min, increasing the temperature to 150 °C at a rate of 5 °C/min, increasing to 220 °C at a rate of 3 °C/min and holding for 30 min. The temperatures of the ion source and MS transfer line were 250 °C and 280 °C, respectively. The scan range was 33–450 *m*/*z* with electron impact (EI) mode at 70 eV.

The NIST 2002 mass spectroscopy library (National Institute of Standards and Technology, Gaithersburg, MD, USA) and the retention times of the authentic standards were used to identify the volatile compounds. Quantification was performed using the peak areas on the total ion chromatogram. The concentrations of the volatile compounds were calculated based on their calibration curves (the relative response rate was greater than 98%), which were built by plotting the area ratio of the target compounds to the internal standard against the concentration ratio [24]. All determinations were performed in triplicate.

### 2.5. Statistical Analysis

All analyses were performed in triplicate, and mean values were calculated. Duncan’s multiple range tests were used to determine significant differences (*p* < 0.05) with SPSS 19.0 software for Windows (SPSS Inc., Chicago, IL, USA). The heatmap was created by Metabo-Analyst 3.0 [25].

## 3. Results and Discussion

### 3.1. Weather Conditions and Physicochemical Parameters of Grape Berries

Growing season rainfall in 2015 was lower than that in 2016 (Table S2). The mean growing season daily temperature and growing season ET_0_ in 2015 were higher than those in 2016. The weather during the years of this study was typical of a semiarid continental climate, which is very suitable for cultivation of Cabernet Sauvignon (*V. vinifera* L.) [26].

The effect of RDI on the physicochemical parameters of grape berries is shown in Table 1. The full irrigation grapes had the highest yield, total acidity (TA), and berry weight and the lowest total soluble solids (TSS) and pH for both years. The RDI-1 and RDI-2 treatment groups had significantly lower yields, berry fresh weights, and TA for both years, which agreed with a previous report [13]. RDI treatments increased the TSS for the two years but not significantly. RDI-1 and RDI-2 treatments significantly increased the pH both years. The RDI treatments reducing the TA and increasing the pH were in accordance with previous reports and could be caused by a reduction in malic acid content [18,27]. The physicochemical parameters of the RDI treatment groups were different for the two years, which might because the growing season rainfall in 2016 was higher than that in 2015 (Table S2).

### 3.2. Amino Acid Profiles of Grape Berries and Wines

Amino acids are the main precursors for secondary metabolites, including flavonoids, in grapes [11], and are also a nitrogen source for yeast during alcoholic fermentation [28,29]. The amino acid profile of grape berries affects the production of polyphenol compounds, ethanol and glycerol [30]. The amino acid profiles of the grape berries and wines in the current experiment are shown in Table 2 and Table 3, respectively. Chromatograms of the amino acid are shown in Figure S1. According to amino acid biosynthesis pathways, 17 of the amino acids present in grape berries and wines can be classified into five families, including serines (Cys, Ser, and Gly), aromatic amino acids (Phe and Trp), aspartates (Ile, Asp, Lys, Met, and Thr), glutamates (Arg, Glu, His, and Pro), and pyruvates (Leu, Ala, and Val) [31].

For the serine family, serine was the most abundant amino acid in grape berries, accounting for 50% of the amino acids from this family (Table 2). RDI-3 treatments significantly increased the levels of serine in both years; lower levels of serine were detected in the RDI-2 treatment group. The highest levels of cysteine were found in 2015 wines produced from RDI-3-treated vines; however, RDI-1 and RDI-2 treatments decreased cysteine values in both vintages of wine (Table 3). Phenylalanine and tryptophan are the main aromatic amino acid families in grape berries and wines. RDI-1 and RDI-3 treatments significantly increased tryptophan levels in the two vintages; however, the lowest tryptophan levels were detected with the RDI-2 treatment for both years (Table 2 and Table 3). The highest levels of phenylalanine were detected in the RDI-3 group for both years, which means that the RDI-3 treatment might give wine more rose, floral, and honey flavors [2]. The concentration of phenylalanine amino acid in berries with the RDI-2 in 2016 was higher compared to the control group. Higher values of aspartic acid in grape berries after RDI-3 treatment might provide more nitrogen for yeast [30,32]. The RDI-1 and RDI-2 treatments decreased the levels of methionine and lysine in berries; however, increased levels of methionine and lysine were found in berries after the RDI-3 treatment (Table 2). Methionine is the precursor of glutathione [29], and the RDI-3 treatment increased the level of methionine in the two vintages wines, which was consistent with a high level of glutathione. In contrast, the RDI-1 and RDI-2 treatments decreased the level of methionine in the two vintages wines, which was consistent with a low level of glutathione (Table 3 and Figure S2). Threonine was the most abundant member of the aspartate amino acid family in berries.

For the glutamate family, proline and arginine were the most abundant amino acids in grape berries (Table 2). Proline plays an important role in enhancing plant tolerance to water deficits [32,33]. The RDI treatments enhanced proline biosynthesis in both vintages, which was consistent with previous reports [32]. Arginine levels were enhanced in berries after the RDI-3 treatment; however, the RDI-2 treatment decreased the arginine levels in both vintages. The levels of arginine were lower in the wines than the berries, which might be due to yeast consuming arginine during fermentation [2]. The leucine and valine levels increased in berries and wines from the RDI-3 treatment for both vintages. The RDI-2 treatment increased the levels of leucine and valine in the 2016 grape berries; however, higher leucine and valine levels were only found in the 2015 wines.

### 3.3. Volatile Compounds Derived from Amino Acids in Grape Berries and Wines

#### 3.3.1. Volatile Compounds Derived from Amino Acids in Grape Berries

**Figure 1:**
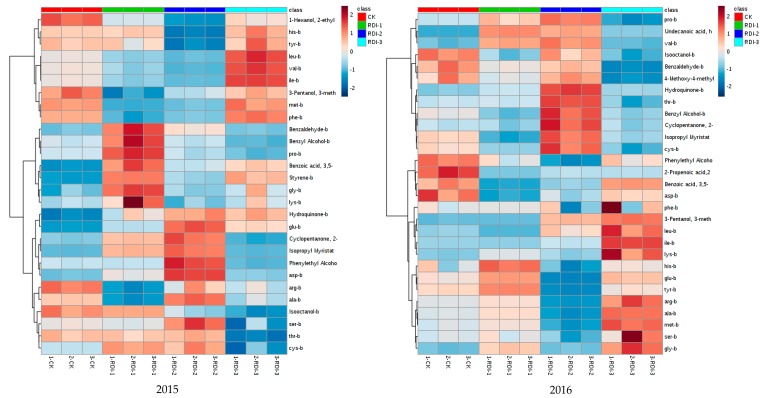
Clustered heatmaps of amino acids and volatile compounds derived from grape berries subjected to different treatments. Data were normalized by a pooled sample from the control group.

The volatile compounds derived from amino acids in the grapes and wines in this experiment are shown in Figure 1 and Table S3. The total amount of aromatic compounds in berries was higher in the RDI-3 treatment group than the other experimental groups (Figure 1 and Table S3). The polyphenol compounds were mainly produced by phenylalanine through the shikimic acid pathway [2]. The results showed that the level of phenylalanine in the berries from the RDI-3 treatment was significantly higher than that in the other treatment groups (Table 2) and, thus, promotes the production of polyphenol [2]. Increased levels of aromatic compounds—such as 3,5-dimethyl-benzoic acid, styrene—and hydroquinone, were detected in berries after the RDI-1 and RDI-2 treatments in 2015, and the increase was significant (Figure 1 and Table S3). Increased levels of aromatic compounds, such as benzyl alcohol, 3,5-dimethyl-benzoic acid, and hydroquinone, were detected in berries after the RDI-3 treatment for both years, and the increase was significant (Figure 1 and Table S3). RDI treatments increased the branched ketone content in berries for both vintages. Interestingly, RDI treatments increased the branched ester content, which could enhance the fruity aroma in berries.

#### 3.3.2. Volatile Compounds Derived from Amino Acids in Wines

The volatile compounds derived from amino acids in wines in this experiment are shown in Table 4. According to volatile compound biosynthesis pathways, the volatile compounds derived from amino acids in wines can be classified into four families, aromatic compounds, branched alcohols, branched ketones, and branched esters. The total amount of aromatic compounds in wines was higher in the RDI-3 treatment group than the other experimental groups (Table 4). The RDI-1 and RDI-2 treatments significantly increased the levels of aromatic compounds, such as benzaldehyde, benzyl alcohol, and phenylethyl alcohol, in the two vintages wines (Table 4). Increased levels of phenylethyl alcohol, 2-methyl-1-propanol and 3-methyl-1-butanol were detected in wines created from the RDI treatment groups, and these compounds might give wines more floral, rose, honey, and fruity flavors [3]. The quantity of branched ketones in wines was significantly higher than that in berries, and RDI treatments increased the branched ketone content in wines for both vintages. Many future works are needed to elucidate the potential effects of different RDI techniques on the accumulation of volatile precursors and amino acids in grapes and wines and their derived volatile compounds.

### 3.4. The Correlation Analysis between Volatile Compounds and Amino Acids in Grape Berries and Wines

The analysis correlation ship between volatile compounds and amino acids in grape berries and wines was shown in Figure S2. The RDI-2 and RDI-3 treatment significantly increased the level of leucine in the two vintages berries and wines (Table 2 and Table 3). The concentration of leucine in berries was positively related to the concentration of leucine in wines (Figure S2). The concentration of leucine was closely related to the concentrations of 2-methyl-1-butyl acetate, benzaldehyde, 3-methyl-1-pentanol and 3-methyl-1-butanol in wines (Figure S2). RDI-3 treatment significantly enhanced the concentrations of arginine, alanine, valine, and isoleucine in both vintages wines and RDI-2 significantly increased the concentrations of valine and isoleucine in both vintages wines (Table 3). The concentrations of arginine, alanine, valine, and isoleucine were positively related to the accumulation of volatile compounds—such as 3,4-dimethyl-benzoic acid, 3-hydroxy-2-butanone, 1-butoxy-2-propanol, 2-methyl-1-butyl acetate, and 3-methyl-1-pentanol—in wines (Figure S2). The accumulation of volatile compounds, after RDI treatments, was closely related to the amino acid concentrations—especially isoleucine, valine, and leucine—in wines.

## 4. Conclusions

The effects of RDI on amino acids and their derived volatiles in Cabernet Sauvignon grapes and wines were evaluated for two consecutive vintages. The RDI-1-treated vines had the lowest yield and berry weight but higher TSS compared with that of the RDI-2 and RDI-3 groups. The RDI-2 and RDI-3 treatments increased the concentrations of amino acids in berries as well as certain volatile components in wines, including 3-methyl-2-pentanol and 3-methyl-1-butanol. The accumulation of volatile compounds in wines is closely related to the content of amino acids, especially isoleucine, valine, and leucine. Overall, the RDI-2 and RDI-3 treatments improved the qualities of grapes and wines based on their amino acid and derived volatile compositions.

## Figures and Tables

**Table 1:** Physicochemical parameters of grape berries from the different treatment groups.

Parameter	RDI-1		RDI-2		RDI-3		Control	
R	2015	2016	2015	2016	2015	2016	2015	2016
Yield (ton/ha)	7.12b	7.70b	7.77ab	7.94b	8.36a	8.58ab	9.06a	9.57a
Weight (g/100 berries)	110.14b	118.18b	105.01c	110.10c	118.38a	124.81a	122.50a	122.70a
Total soluble solids (Brix)	23.92a	23.45a	23.53ab	23.95a	23.56ab	23.31a	22.30b	23.26a
Titratable acidity (g/L tartaric acid)	4.42b	3.25b	4.81ab	3.14b	5.12ab	3.51a	5.46a	3.71a
pH	3.90a	4.13b	3.72b	4.31a	3.69c	3.92c	3.67c	3.86c
Midday stem water potential from fruit set to harvest (MPa)	−0.56	−0.58	−0.49	−0.52	−0.38	−0.43	−0.30	−0.34

Different letters in the row indicate significant differences (*p* < 0.05) among treatments for the same vintage.

**Table 2:** Amino acid concentrations (mg/L) in grapes from untreated (CK) and treated vineyards.

Family	Amino Acid	2015				2016			
		RDI-1	RDI-2	RDI-3	CK	RDI-1	RDI-2	RDI-3	CK
Serine	Cys	5.96 ± 1.81a	7.92 ± 1.13a	10.78 ± 0.05a	8.55 ± 0.40a	5.76 ± 0.07a	6.70 ± 0.69a	8.43 ± 0.49a	6.17 ± 0.84a
	Ser	35.26 ± 1.21b	38.56 ± 1.02ab	41.24 ± 3.01a	39.25 ± 1.78ab	33.58 ± 0.24ab	28.543 ± 2.01b	42.75 ± 2.06a	30.52 ± 0.78b
	Gly	3.18 ± 0.45c	3.40 ± 0.20c	7.26 ± 0.43a	7.82 ± 0.10b	4.92 ± 0.01ab	2.08 ± 0.29b	8.96 ± 0.18a	2.77 ± 0.11b
Aromatic amino acids	Phe	15.58 ± 0.85c	16.85 ± 0.45c	26.41 ± 0.77a	20.00 ± 0.88b	17.12 ± 1.39b	15.26 ± 1.33c	18.71 ± 0.97a	16.18 ± 0.90bc
	Trp	11.24 ± 0.48ab	6.95 ± 0.21c	15.49 ± 0.89a	9.54 ± 0.32b	15.88 ± 0.54a	6.21 ± 0.81c	14.39 ± 0.31a	8.82 ± 0.35b
Aspartate	Ile	8.61 ± 1.04c	7.62 ± 0.64c	17.62 ± 1.05a	12.34 ± 0.62b	10.65 ± 0.95b	12.06 ± 0.95b	19.17 ± 0.92a	8.14 ± 0.98b
	Asp	20.16 ± 0.86b	25.31 ± 0.73a	25.08 ± 0.76a	21.55 ± 0.56b	18.99 ± 1.61bc	18.28 ± 0.79c	23.16 ± 0.44a	21.34 ± 1.08ab
	Lys	2.51 ± 0.81b	2.45 ± 0.21b	5.21 ± 0.21a	3.45 ± 0.12b	3.01 ± 0.32b	1.98 ± 0.08b	4.30 ± 0.05a	2.68 ± 0.0.31b
	Met	1.64 ± 0.04c	1.58 ± 0.15c	4.95 ± 0.75a	3.21 ± 0.09b	2.41 ± 0.21b	1.02 ± 0.01d	5.01 ± 0.02a	1.95 ± 0.04c
	Thr	47.62 ± 2.68b	51.26 ± 1.53b	57.24 ± 0.92a	57.32 ± 1.46a	47.76 ± 1.23ab	47.85 ± 0.69ab	49.56 ± 0.60a	44.55 ± 3.15b
Glutamate	Arg	330.19 ± 2.61b	336.47 ± 3.13b	354.54 ± 1.52a	346.57 ± 1.26a	320.54 ± 1.01b	308.47 ± 0.30c	334.85 ± 6.09a	317.55 ± 4.37bc
	Glu	69.74 ± 1.53c	82.42 ± 2.13b	89.85 ± 0.84a	70.45 ± 0.17c	64.45 ± 2.01a	40.54 ± 0.99c	66.37 ± 0.66a	54.25 ± 1.45b
	His	16.32 ± 1.04c	15.21 ± 0.98c	27.39 ± 2.30a	21.52 ± 1.51b	22.17 ± 1.64ab	15.05 ± 0.95c	25.45 ± 2.01a	18.95 ± 1.01bc
	Pro	195.77 ± 1.61a	139.45 ± 2.59b	122.26 ± 10.36b	105.12 ± 5.526c	155.49 ± 0.82a	145.46 ± 2.04b	119.89 ± 3.80c	115.64 ± 1.84c
Pyruvate	Leu	15.95 ± 1.58c	24.58 ± 1.08b	31.05 ± 22.85a	21.95 ± 2.84b	17.82 ± 1.84b	15.24 ± 2.01b	29.54 ± 2.54a	14.85 ± 0.54b
	Ala	35.78 ± 4.18b	63.83 ± 3.69a	74.70 ± 59.24a	67.94 ± 0.67a	51.65 ± 2.75b	31.20 ± 0.87d	71.03 ± 2.74a	43.57 ± 0.72c
	Val	13.51 ± 1.59c	12.95 ± 1.52c	27.62 ± 2.81a	20.12 ± 0.43b	22.57 ± 1.24a	20.14 ± 0.76a	24.32 ± 2.31a	15.89 ± 0.81b

Different letters in the row indicate significant differences (*p* < 0.05) among treatments for the same vintage.

**Table 3:** Amino acid concentrations (mg/L) in wines from untreated (CK) and treated vineyards.

Family	Amino Acid	2015				2016			
		RDI-1	RDI-2	RDI-3	CK	RDI-1	RDI-2	RDI-3	CK
Serine	Cys	3.18 ± 0.13b	4.12 ± 0.06b	7.22 ± 0.13a	6.41 ± 0.26ab	2.94 ± 0.05b	3.21 ± 0.67b	5.99 ± 0.02a	5.96 ± 0.19a
	Ser	1.40 ± 0.03c	3.75 ± 0.43b	6.21 ± 0.61a	2.11 ± 0.15c	1.05 ± 0.05c	5.66 ± 0.43b	8.82 ± 0.42a	4.30 ± 0.40b
	Gly	2.32 ± 0.77c	4.62 ± 0.93b	6.32 ± 0.42a	3.05 ± 0.38c	2.74 ± 0.10c	4.41 ± 0.69c	5.83 ± 0.06a	3.75 ± 0.77b
Aromatic amino acids	Trp	2.06 ± 0.22c	3.34 ± 0.37b	4.07 ± 0.75a	1.73 ± 0.51c	2.55 ± 0.50b	4.04 ± 0.37b	5.51 ± 0.74a	2.51 ± 0.54c
	Phe	1.88 ± 0.02c	2.62 ± 0.09b	4.14 ± 0.52a	1.03 ± 0.04c	2.05 ± 0.22c	1.51 ± 0.33c	3.67 ± 0.12a	2.62 ± 0.11b
Aspartate	Ile	0.24 ± 0.06c	2.03 ± 0.26b	3.64 ± 0.17a	1.83 ± 0.29c	0.65 ± 0.16d	1.63 ± 0.05c	2.21 ± 0.05a	1.46 ± 0.15b
	Lys	1.59 ± 0.01c	2.08 ± 0.12b	3.7 ± 0.07a	1.86 ± 0.18c	1.04 ± 0.17b	2.91 ± 0.34ab	3.29 ± 0.09a	1.69 ± 0.15b
	Met	0.4 ± 0.03c	0.49 ± 0.09c	1.56 ± 0.06a	0.69 ± 0.04b	0.55 ± 1.09c	0.51 ± 0.08c	1.82 ± 0.04a	0.66 ± 0.07b
	Asp	2.79 ± 0.21c	3.63 ± 0.15b	4.6 ± 0.29a	2.07 ± 0.10c	1.06 ± 0.09c	3.55 ± 0.18a	3.19 ± 0.41a	1.89 ± 0.26b
	Thr	1.12 ± 0.07c	2.78 ± 0.14b	2.12 ± 0.07a	1.05 ± 0.02c	1.48 ± 0.14c	1.84 ± 0.02c	2.4 ± 0.15a	1.85 ± 0.20b
Glutamate	Pro	16.24 ± 0.89c	15.57 ± 2.39b	14.51 ± 3.48a	9.47 ± 0.55c	13.49 ± 1.14c	12.74 ± 1.51b	13.47 ± 2.60a	10.06 ± 1.10c
	His	1.43 ± 0.14a	1.59 ± 0.04a	1.91 ± 0.07a	0.55 ± 0.02b	1.56 ± 0.05b	1.86 ± 0.01b	2.51 ± 0.06a	0.56 ± 0.01c
	Arg	12.59 ± 1.98b	10.52 ± 1.12c	15.00 ± 0.16a	10.75 ± 0.91c	15.06 ± 1.51b	13.28 ± 1.00b	17.87 ± 1.73a	14.11 ± 1.28b
	Glu	4.65 ± 1.73b	2.44 ± 0.53c	7.74 ± 0.17a	5.74 ± 0.92ab	3.41 ± 2.13c	2.44 ± 0.13c	8.66 ± 1.04a	6.26 ± 1.07b
Pyruvate	Leu	5.4 ± 0.79c	8.67 ± 0.59b	10.66 ± 1.28a	6.76 ± 0.51c	6.09 ± 0.14c	7.06 ± 0.40b	9.23 ± 0.26a	5.88 ± 0.54c
	Ala	5.72 ± 0.28c	6.13 ± 0.48b	9.49 ± 0.94a	4.31 ± 0.14c	6.91 ± 1.36c	7.11 ± 1.55c	10.55 ± 0.43a	8.51 ± 0.96b
	Val	1.27 ± 0.06c	2.45 ± 0.18b	4.71 ± 0.45a	1.1 ± 0.12c	1.09 ± 0.71b	1.33 ± 0.05b	3.78 ± 0.49a	1.08 ± 0.41b

Different letters in the row indicate significant differences (*p* < 0.05) among treatments for the same vintage.

**Table 4:** Volatile compound concentrations (μg/L) in wines from untreated (CK) and treated vineyards.

Compounds	2015				2016			
	RDI-1	RDI-2	RDI-3	CK	RDI-1	RDI-2	RDI-3	CK
Benzeneacetic acid, methyl ester	3.74 ± 0.23a	1.62 ± 0.10c	4.2 ± 0.26a	2.56 ± 0.16b	1.43 ± 0.09c	1.72 ± 0.10c	4.05 ± 0.24a	2.54 ± 0.15b
1-propenyl-benzene	0.52 ± 0.03c	1.02 ± 0.06a	0.77 ± 0.05b	0.8 ± 0.05b	1.29 ± 0.08b	1.89 ± 0.11b	1.11 ± 0.07a	1.33 ± 0.08b
Benzaldehyde	2.86 ± 0.18b	2.98 ± 0.18b	4.96 ± 0.31a	1.87 ± 0.12c	3.17 ± 0.19b	2.11 ± 0.13c	5.37 ± 0.32a	2.97 ± 0.18bc
3,4-Dimethyl-benzoic acid	2.53 ± 0.16c	4.77 ± 0.3a	3.37 ± 0.21bc	3.6 ± 0.22b	4.99 ± 0.3a	5.13 ± 0.31a	4.52 ± 0.27a	4.68 ± 0.28a
Styrene	2.76 ± 0.17ab	3 ± 0.19a	2.65 ± 0.16ab	2.29 ± 0.14b	5.34 ± 0.32a	6.75 ± 0.4a	6.43 ± 0.38a	5.6 ± 0.33a
Benzyl alcohol	0.48 ± 0.03b	0.85 ± 0.05a	0.89 ± 0.06a	0.63 ± 0.04b	0.65 ± 0.04ab	0.61 ± 0.04b	0.79 ± 0.05a	0.57 ± 0.03b
Phenylethyl alcohol	56.43 ± 3.5a	52.1 ± 3.23a	55.35 ± 3.43a	49.57 ± 3.07a	58.27 ± 3.48a	57.16 ± 3.41a	55.83 ± 3.33a	53.71 ± 3.21a
Furfural	2.46 ± 0.15a	2.31 ± 0.14a	2.32 ± 0.14a	2.41 ± 0.15a	nd^b^	nd	nd	nd
3-Furaldehyde	0.27 ± 0.02b	0.61 ± 0.04a	0.56 ± 0.03a	0.16 ± 0.01b	nd	nd	nd	nd
2,3,4,6-Tetramethylphenol	1.85 ± 0.11a	0.85 ± 0.05b	1.55 ± 0.1a	1.88 ± 0.12a	2.6 ± 0.16ab	2.65 ± 0.16ab	3.21 ± 0.19a	2.36 ± 0.14b
**Total amount of aromatic compounds**	73.89 ± 4.58a	70.11 ± 4.35a	76.62 ± 4.75a	65.78 ± 4.08a	78.19 ± 4.67a	78.45 ± 4.68a	81.87 ± 4.89a	74.26 ± 4.43a
Dihydro-3-methyl-2,5-furandione	nd	nd	nd	nd	0.45 ± 0.03ab	0.42 ± 0.03b	0.55 ± 0.03a	0.49 ± 0.03ab
2-Methyl-1-propanol	12 ± 0.74a	11.34 ± 0.7ab	10.57 ± 0.66ab	9.25 ± 0.57b	22.74 ± 1.36a	16.22 ± 0.97b	22.2 ± 1.33a	16.16 ± 0.96b
3-Methyl-1-pentanol	2.73 ± 0.17c	5.41 ± 0.34a	4.41 ± 0.27ab	3.42 ± 0.21bc	nd	nd	nd	nd
3-Methyl-1-butanol	192.09 ± 11.91a	203.06 ± 12.59a	222.58 ± 13.8a	202.78 ± 12.57a	315.86 ± 18.85ab	305.71 ± 18.25ab	353.97 ± 21.13a	275.11 ± 16.42b
3-Methyl-2-pentanol	0.86 ± 0.05b	0.59 ± 0.04c	1.21 ± 0.07a	0.98 ± 0.06ab	1.28 ± 0.08bc	1.08 ± 0.06c	1.63 ± 0.1a	1.55 ± 0.09ab
2-Propanol, 1-butoxy-	0.54 ± 0.03b	1.31 ± 0.08a	0.55 ± 0.03b	0.35 ± 0.02b	nd	nd	nd	nd
**Total amount of branched alcohols**	208.22 ± 12.91a	221.72 ± 13.75a	239.31 ± 14.84a	216.79 ± 13.44a	339.87 ± 20.29ab	323.01 ± 19.28ab	377.8 ± 22.55a	292.82 ± 17.48b
2-Butanone, 3-hydroxy-	0.95 ± 0.06b	0.42 ± 0.03c	1.74 ± 0.11a	0.88 ± 0.05b	0.88 ± 0.05b	1.43 ± 0.09a	1.38 ± 0.08a	1.25 ± 0.07a
**Total amount of branched ketones**	0.95 ± 0.06b	0.42 ± 0.03c	1.74 ± 0.11a	0.88 ± 0.05b	0.88 ± 0.05b	1.43 ± 0.09a	1.38 ± 0.08a	1.25 ± 0.07a
2-Methyl-1-butyl acetate	10.5 ± 0.03b	10.8 ± 0.05a	10.7 ± 0.04a	10.53 ± 0.03b	10.78 ± 0.05b	11.07 ± 0.06a	10.93 ± 0.06ab	10.94 ± 0.06ab
Phenylmethyl acetate	10.24 ± 0.01a	10.14 ± 0.01b	10.24 ± 0.01a	10.15 ± 0.01b	10.33 ± 0.02b	10.24 ± 0.01c	10.41 ± 0.02a	10.27 ± 0.02bc
Phenethyl acetate	11.34 ± 0.01b	12.98 ± 0.03a	11.47 ± 0.02b	11.59 ± 0.01b	11.29 ± 0.05b	12.85 ± 0.06a	11.39 ± 0.01b	11.41 ± 0.02b
Isoamyl octanoate	15.36 ± 0.03b	19.21 ± 0.01a	19.38 ± 0.11a	20.62 ± 0.06a	14.98 ± 0.07b	20.35 ± 0.05a	21.68 ± 0.09a	21.92 ± 0.03a
Isoamyl hexanoate	12.35 ± 0.06b	15.34 ± 0.13a	15.03 ± 0.1a	14.95 ± 0.11a	12.68 ± 0.05b	16.92 ± 0.19a	16.28 ± 0.14a	15.62 ± 0.02a
**Total amount of branched esters**	59.79 ± 0.05b	68.47 ± 0.06a	66.82 ± 0.06a	67.84 ± 0.04a	60.06 ± 0.07b	71.43 ± 0.08a	70.69 ± 0.08a	70.16 ± 0.07a

Different letters in the row indicate significant differences (*p* < 0.05) among treatments for the same vintage. nd: not detected.

## Supplementary Materials

The following are available online: Figure S1–S2, Table S1–S3.
