# Supplementary material for: Effects of Regulated Deficit Irrigation on Amino Acid Profiles and Their Derived Volatile Compounds in Cabernet Sauvignon (Vitis vinifera L.) Grapes and Wines
Source: Molecules. 2018 Aug 9;23(8):1983. doi: 10.3390/molecules23081983 (PMC6222330; doi:10.3390/molecules23081983)
Supplement: Supplementary file 1 [file molecules-23-01983-s001.pdf]

Effects of regulated deficit irrigation on amino acid profiles and their derived volatile compounds in Cabernet Sauvignon (*Vitis vinifera* L.) grapes and wines

Yan-lun Ju[a+], Guo-qian Xu[b+], Xiao-feng Yue[a], Xian-fang Zhao[a], Ting-yao Tu[a], Jun-xiang Zhang[b*] and Yu-lin Fang[a,c*]

[a] College of Enology, Northwest A&F University, Yangling, Shaanxi, 712100 China; juyanlun2016@nwsuaf.edu.cn (Y.-L.J.); yuexiaofeng@nwsuaf.edu.cn (X.-F.Y.); zhaoxianfang@nwsuaf.edu.cn (X.-F.Z.); tuty@lzlj.com (T.-Y.T.)

[b] Ningxia Grape and Wine Research Institute, Ningxia University, Yinchuan, Ningxia, 750000 China; xugql@163.com (G.X.); zhangjunxiang@126.com (J.-X.Z.)

[c] Shaanxi Engineering Research Center for Viti-Viniculture, Yangling, Shaanxi, 712100 China

+ These authors equally contributed to this work.

* Correspondence: fangyulin@nwsuaf.edu.cn; Tel.: +86-29-87091874, Fax: 029-87092233

Supplementary Materials:

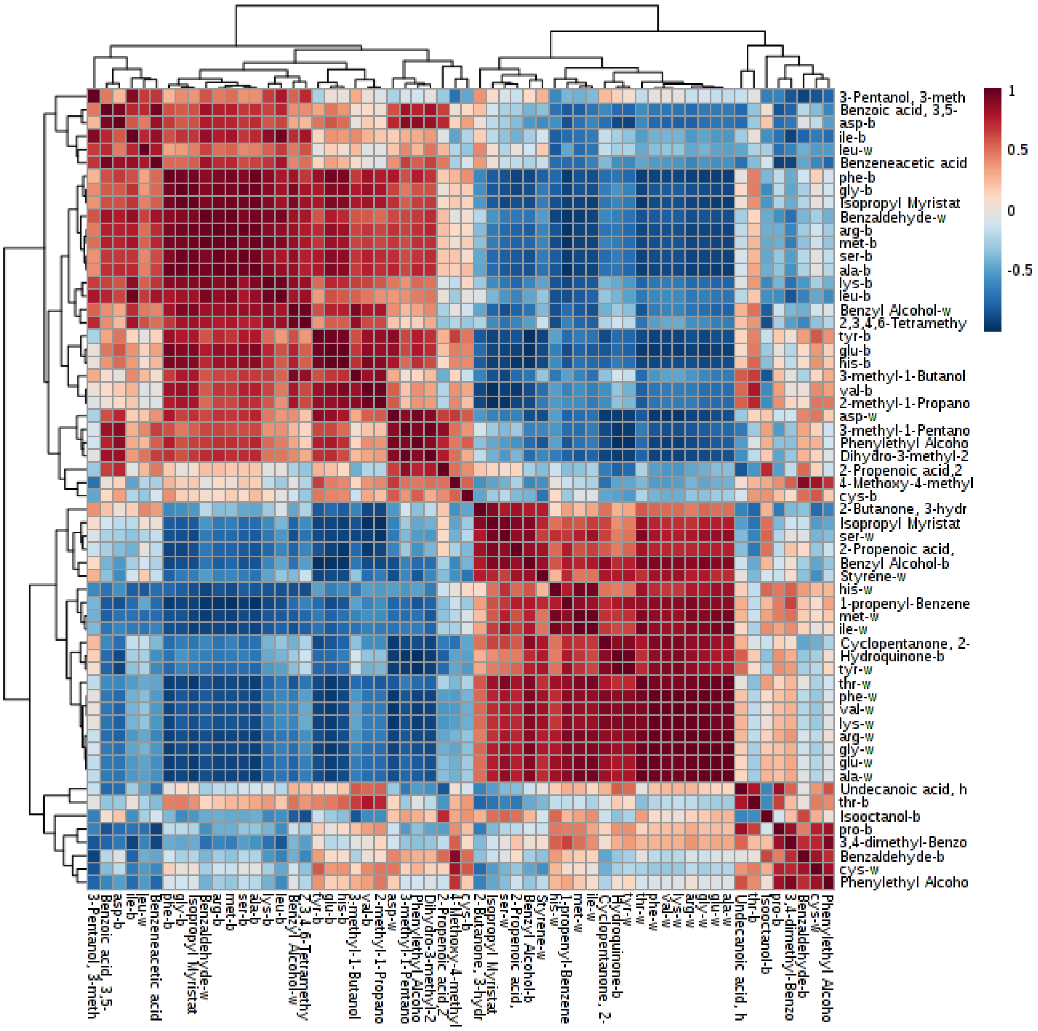

Figure S1. Correlation analysis of amino acids and volatile compounds in this study from grapes and wines with different treatments. Data was normalized by a pooled sample from control groups.

Figure S2

Standard

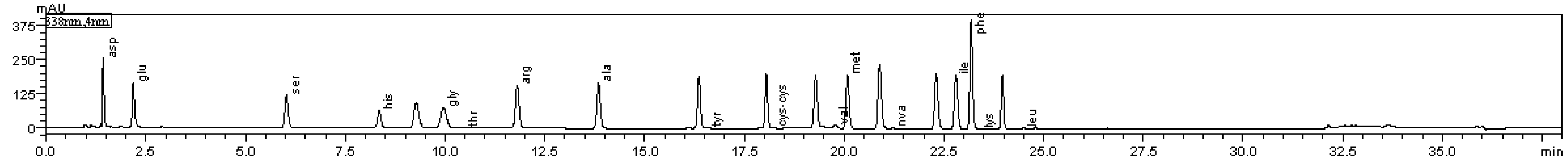

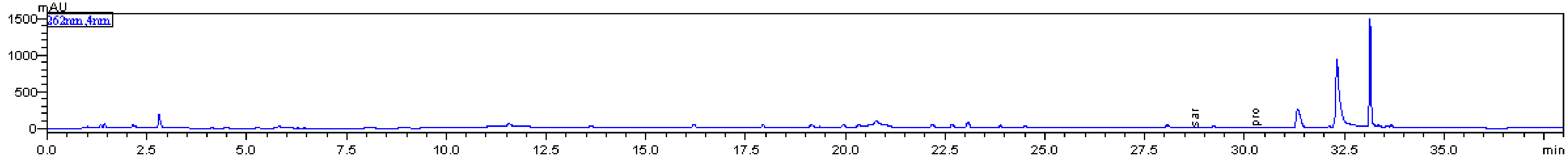

A

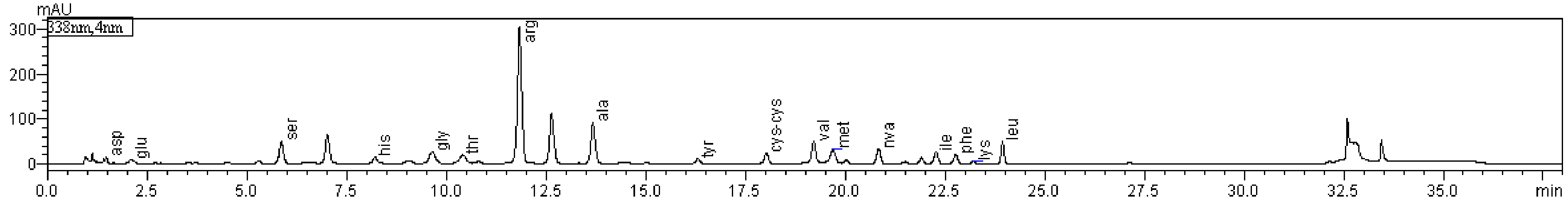

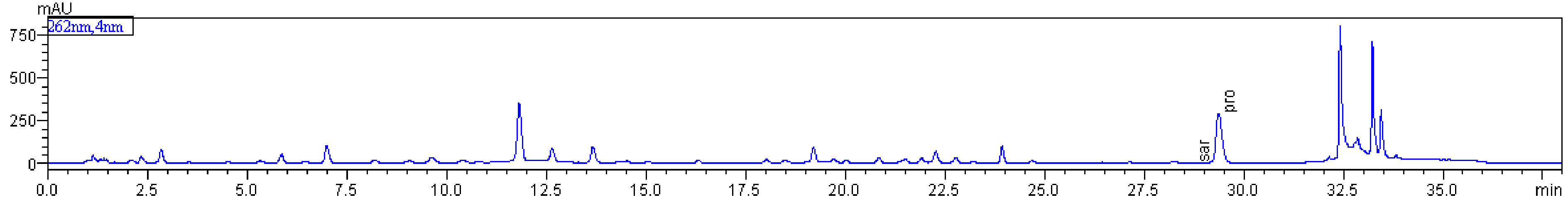

B

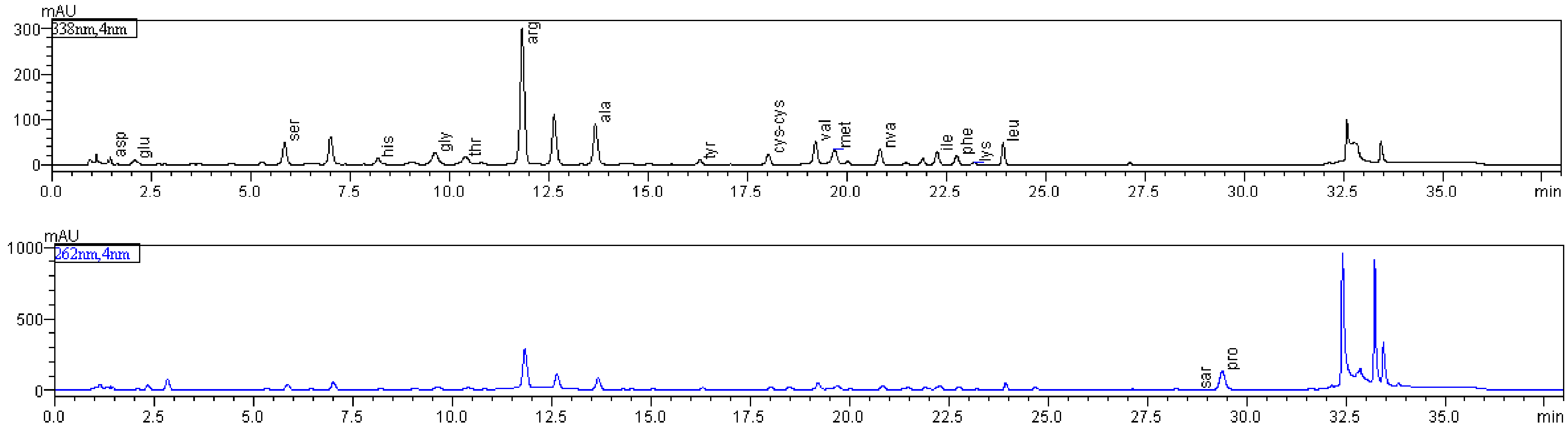
mAU
338nm,4nm
300
200
100
0
asp
glu
ser
his
gly
thr
arg
ala
tyr
cys-cys
val
met
nva
ile
phe
lys
leu
0.0 2.5 5.0 7.5 10.0 12.5 15.0 17.5 20.0 22.5 25.0 27.5 30.0 32.5 35.0 min
mAU
262nm,4nm
1000
500
0
sar
pro
0.0 2.5 5.0 7.5 10.0 12.5 15.0 17.5 20.0 22.5 25.0 27.5 30.0 32.5 35.0 min

C

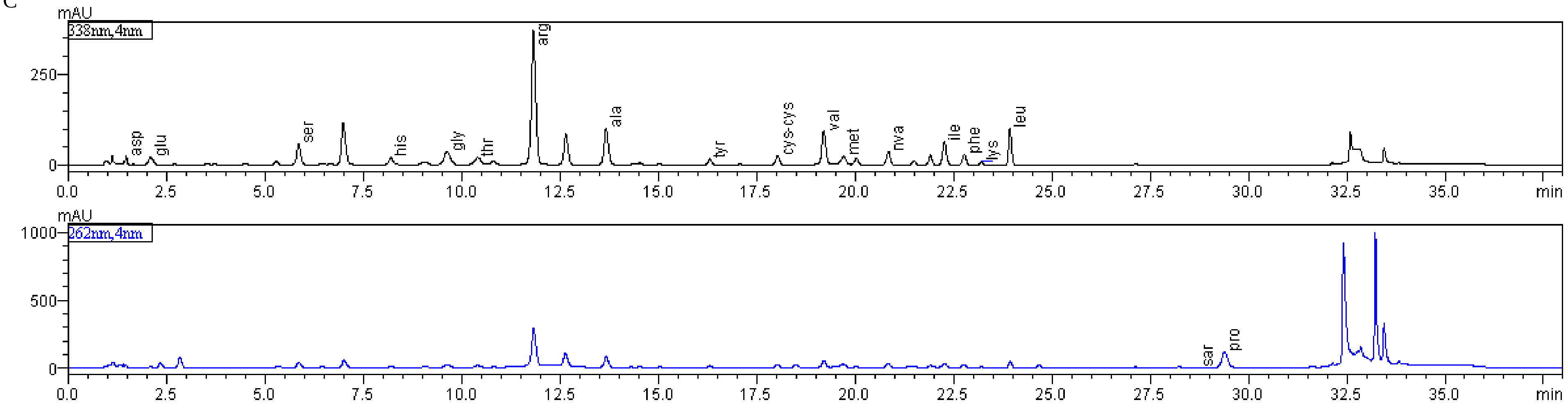
mAU
338nm,4nm
250
0
asp
glu
ser
his
gly
thr
arg
ala
tyr
cys-cys
val
met
nva
ile
phe
lys
leu
0.0 2.5 5.0 7.5 10.0 12.5 15.0 17.5 20.0 22.5 25.0 27.5 30.0 32.5 35.0 min
mAU
262nm,4nm
1000
500
0
sar
pro
0.0 2.5 5.0 7.5 10.0 12.5 15.0 17.5 20.0 22.5 25.0 27.5 30.0 32.5 35.0 min

D

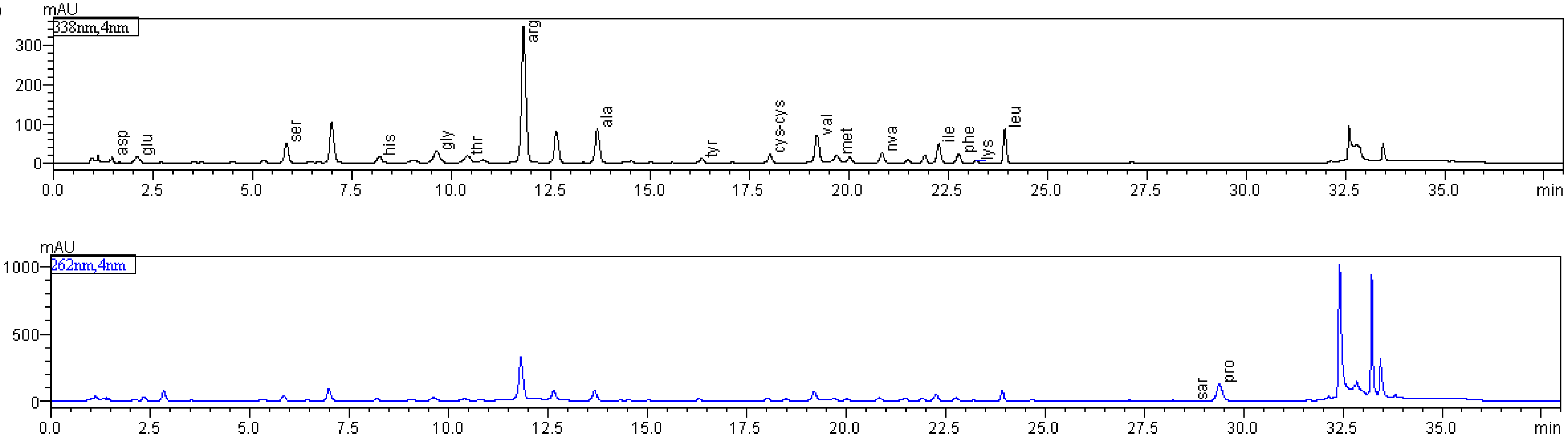
mAU
338nm,4nm
300
200
100
0
asp
glu
ser
his
gly
thr
arg
ala
tyr
cys-cys
val
met
nva
ile
phe
lys
leu
0.0
2.5
5.0
7.5
10.0
12.5
15.0
17.5
20.0
22.5
25.0
27.5
30.0
32.5
35.0
min
mAU
262nm,4nm
1000
500
0
sar
pro
min

E

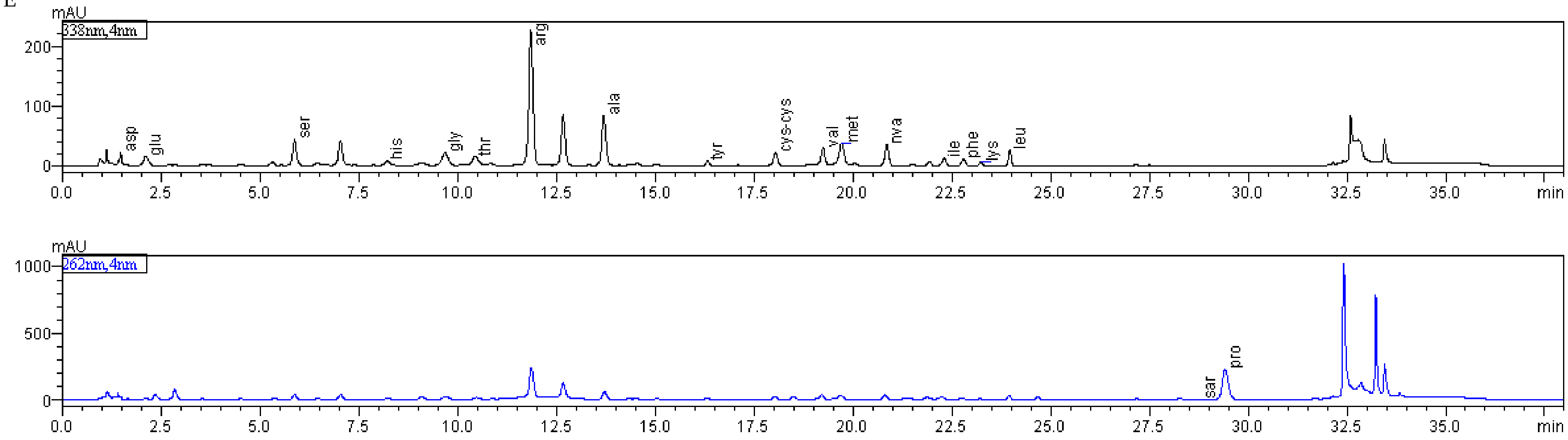
mAU
338nm,4nm
200
100
0
asp
glu
ser
his
gly
thr
arg
ala
tyr
cys-cys
val
met
nva
ile
phe
lys
leu
0.0
2.5
5.0
7.5
10.0
12.5
15.0
17.5
20.0
22.5
25.0
27.5
30.0
32.5
35.0
min
mAU
262nm,4nm
1000
500
0
sar
pro
min

F

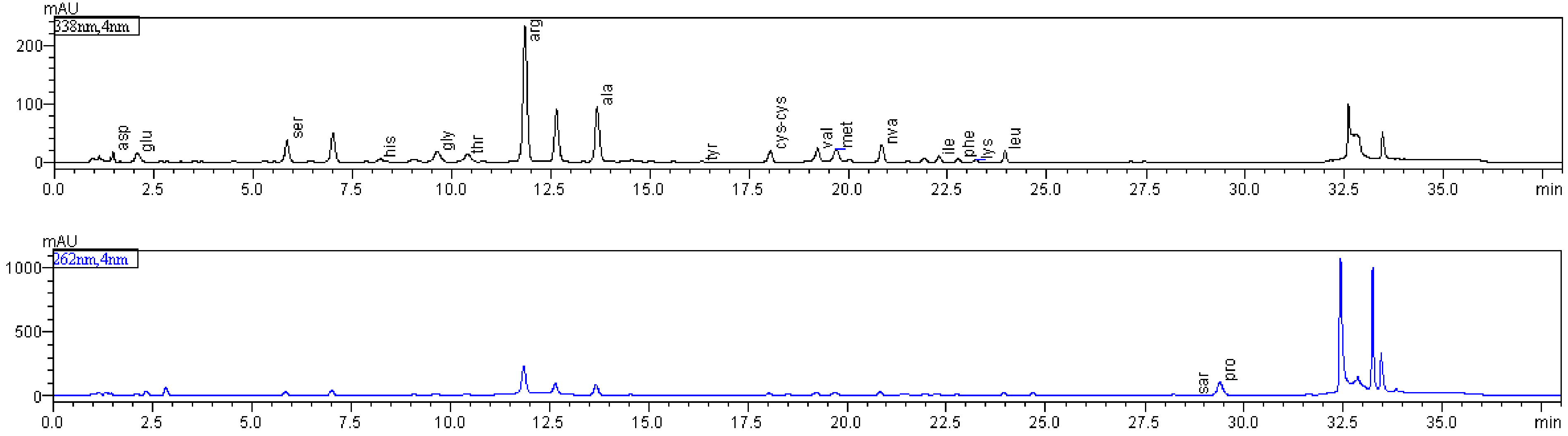
mAU
338nm,4nm
200
100
0
asp
glu
ser
his
gly
thr
arg
ala
tyr
cys-cys
val
met
nva
ile
phe
lys
leu
0.0
2.5
5.0
7.5
10.0
12.5
15.0
17.5
20.0
22.5
25.0
27.5
30.0
32.5
35.0
min
mAU
262nm,4nm
1000
500
0
sar
pro
min

G

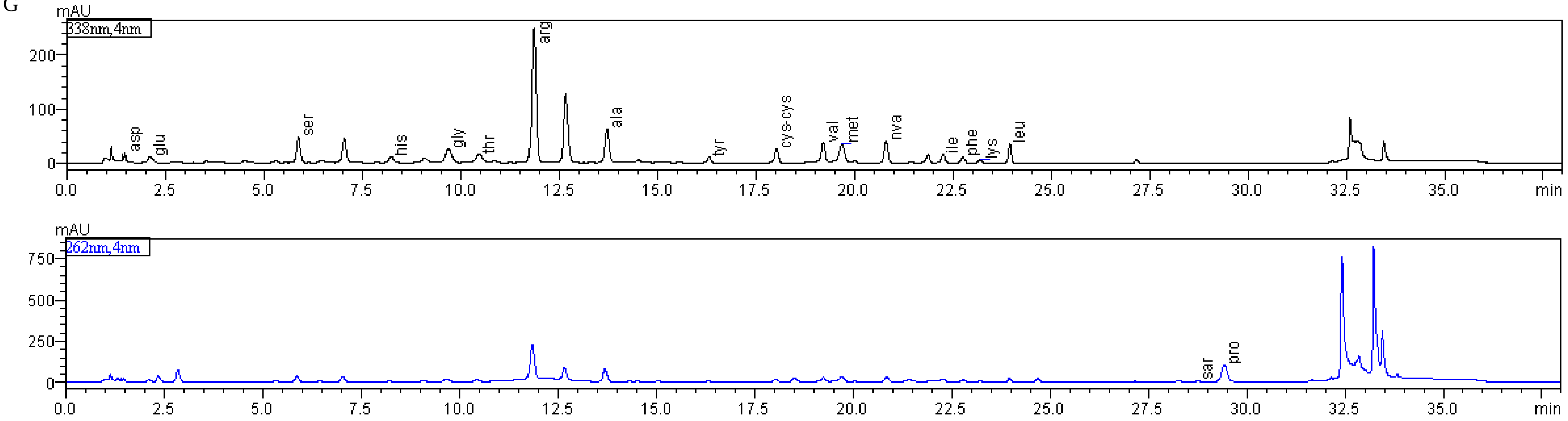
mAU
338nm,4nm
200
100
0
asp
glu
ser
his
gly
thr
arg
ala
tyr
cys-cys
val
met
nva
ile
phe
lys
leu
0.0
2.5
5.0
7.5
10.0
12.5
15.0
17.5
20.0
22.5
25.0
27.5
30.0
32.5
35.0
min
mAU
262nm,4nm
750
500
250
0
sar
pro
min

H

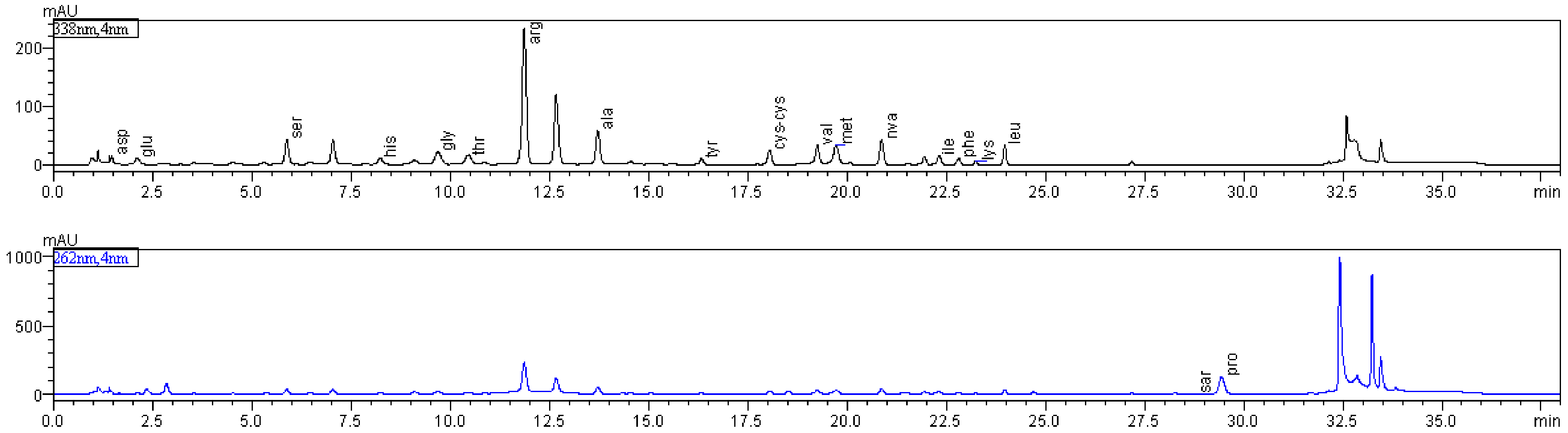
mAU
338nm,4nm
asp
glu
ser
his
gly
thr
arg
ala
tyr
cys-cys
val
met
nva
ile
phe
lys
leu
mAU
262nm,4nm
sar
pro
min

a

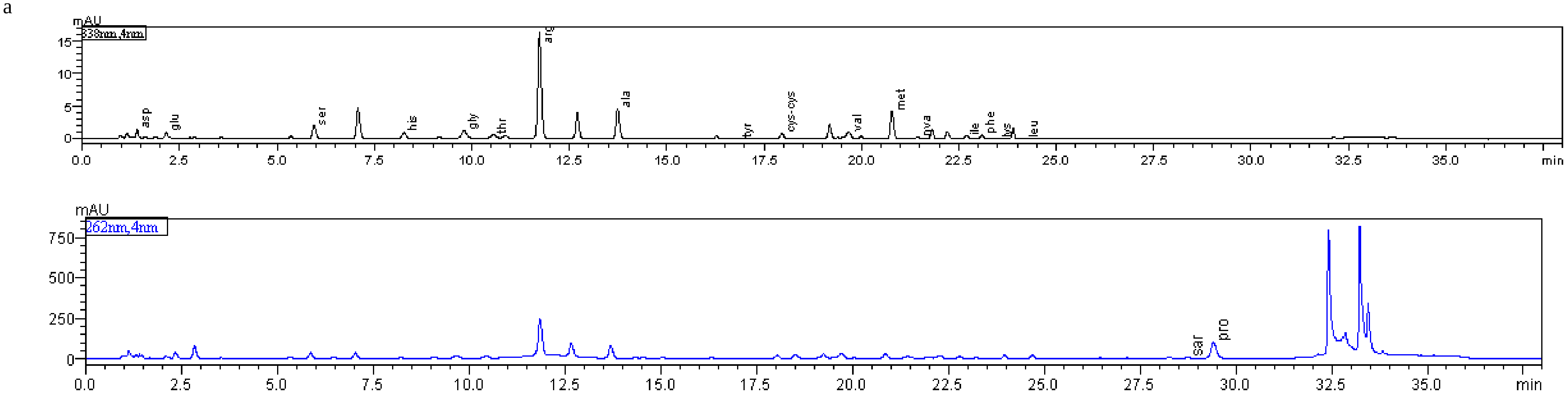
mAU
338nm,4nm
asp
glu
ser
his
gly
thr
arg
ala
tyr
cys-cys
val
met
nva
ile
phe
lys
leu
mAU
262nm,4nm
sar
pro
min

b

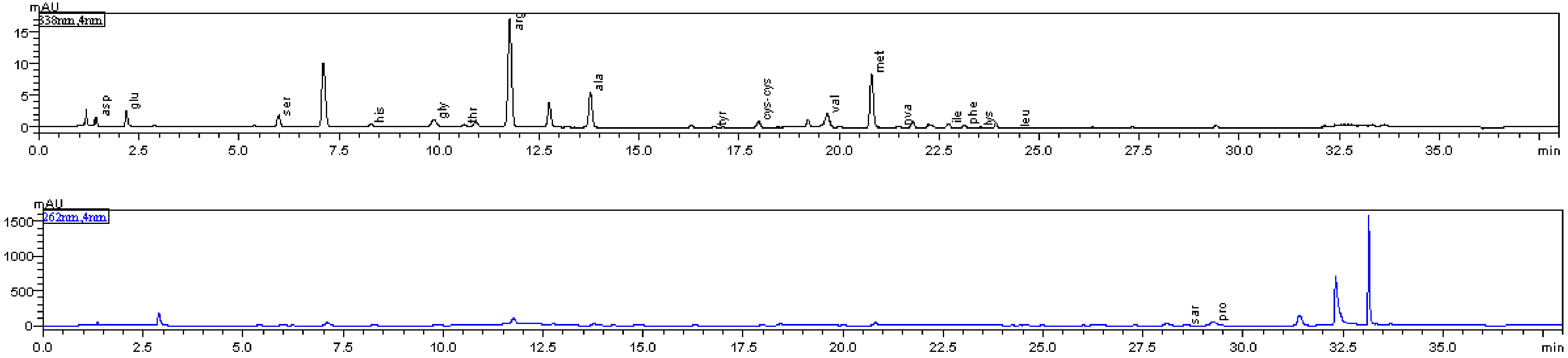

mAU
338nm,4nm
asp
glu
ser
his
gly
thr
arg
ala
tyr
cys-cys
val
met
nva
ile
phe
lys
leu
min
mAU
262nm,4nm
sar
pro
min

c

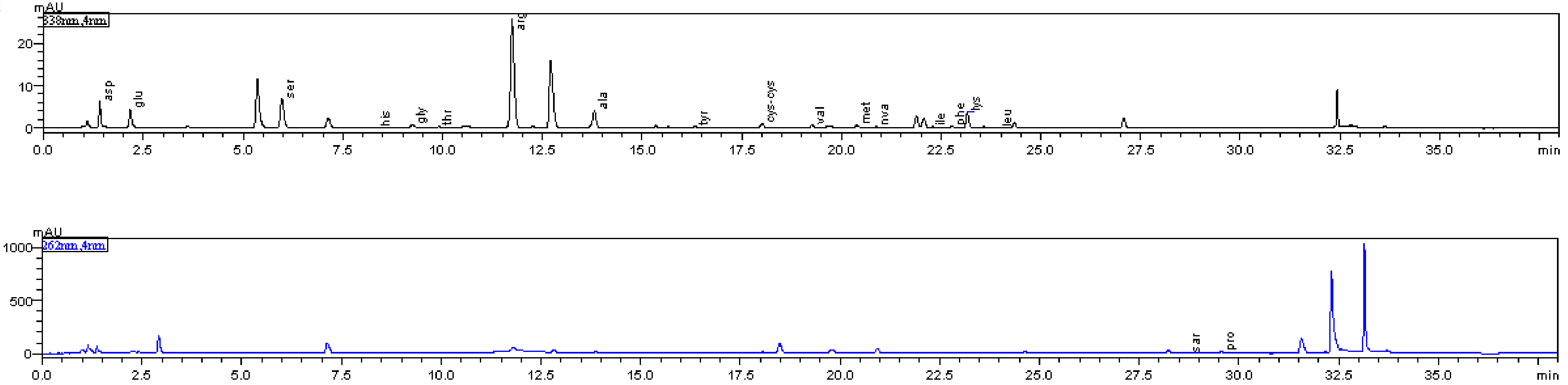

mAU
338nm,4nm
asp
glu
ser
his
gly
thr
arg
ala
tyr
cys-cys
val
met
nva
ile
phe
lys
leu
min
mAU
262nm,4nm
sar
pro
min

d

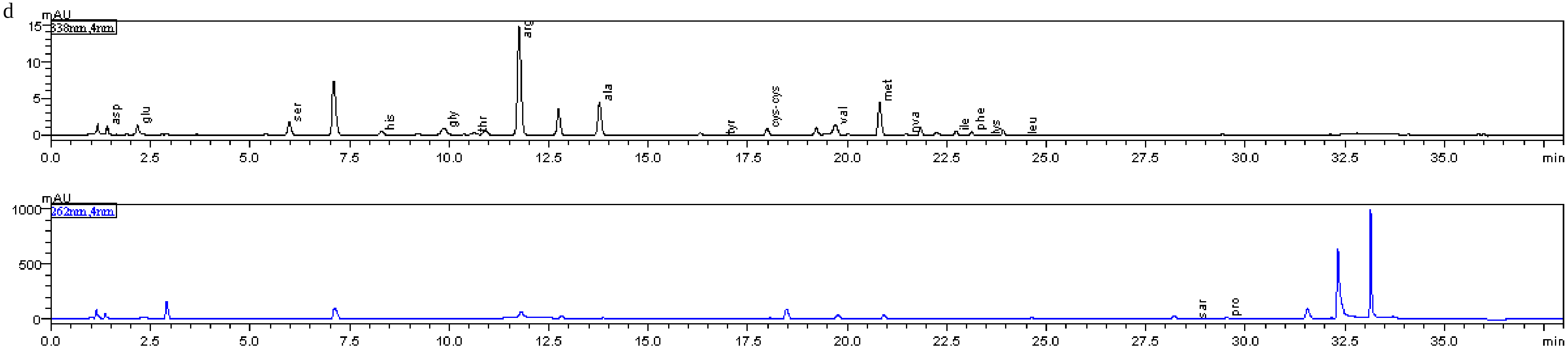
mAU
338nm,4nm
asp
glu
ser
his
gly
thr
arg
ala
tyr
cys-cys
val
met
nva
ile
phe
lys
leu
min
262nm,4nm
sar
pro

e

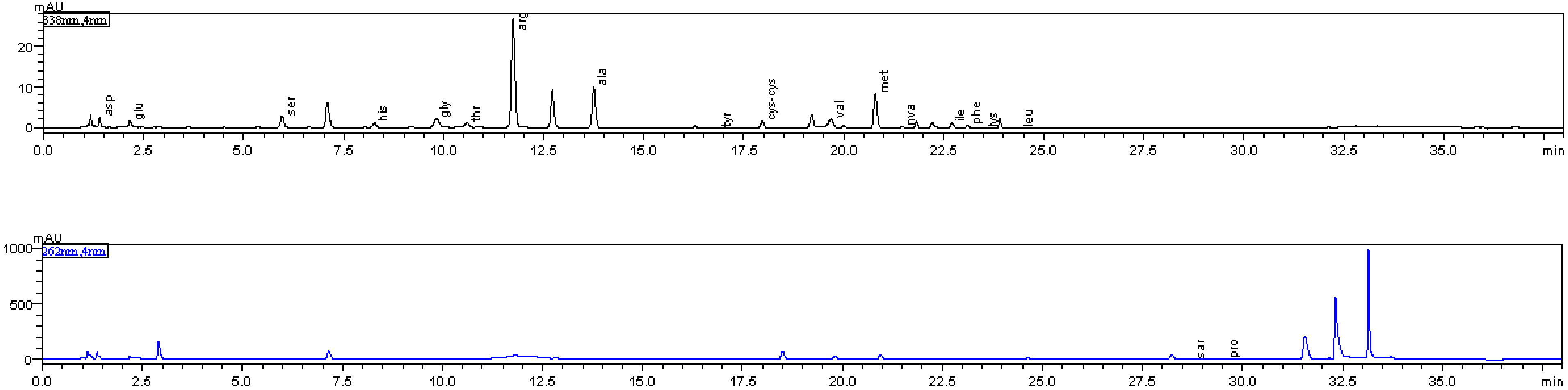
mAU
338nm,4nm
asp
glu
ser
his
gly
thr
arg
ala
tyr
cys-cys
val
met
nva
ile
phe
lys
leu
min
262nm,4nm
sar
pro

f

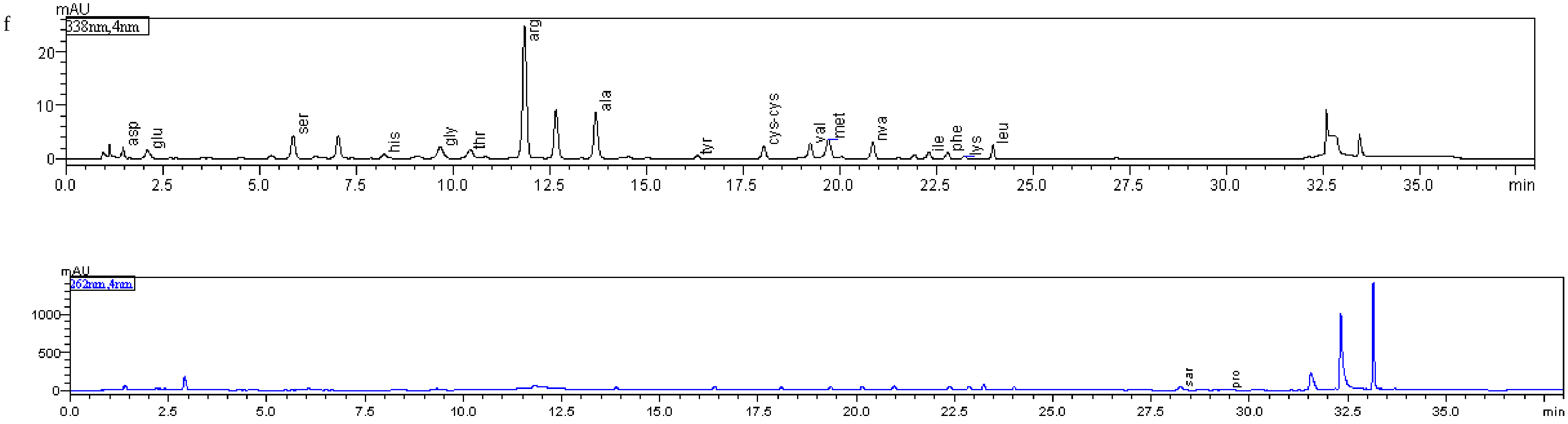
mAU
338nm,4nm
asp
glu
ser
his
gly
thr
arg
ala
tyr
cys-cys
val
met
nva
ile
phe
lys
leu
min
262nm,4nm
sar
pro

g

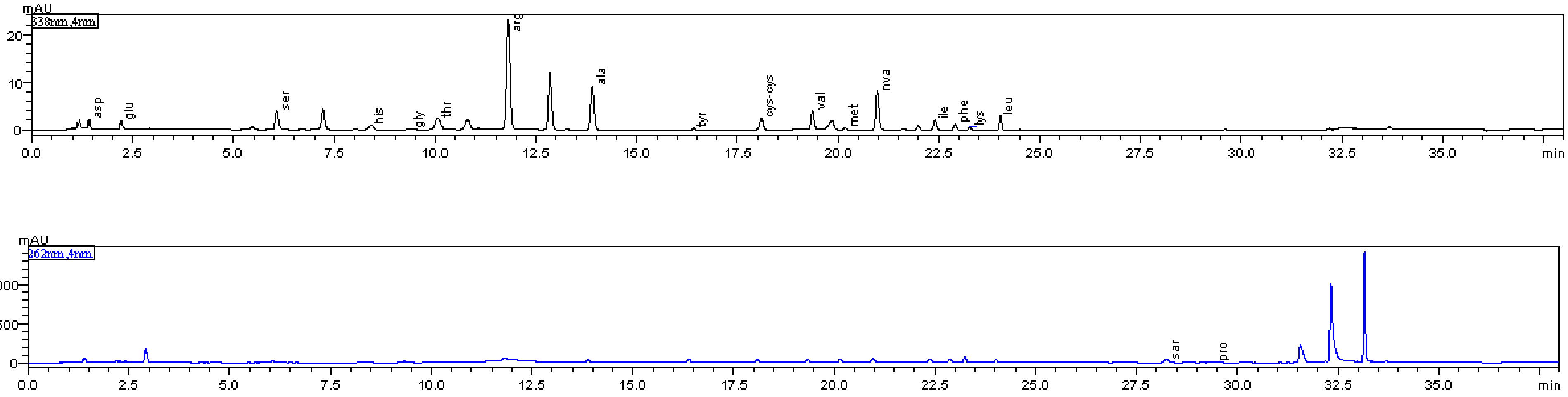
mAU
338nm,4nm
asp
glu
ser
his
gly
thr
arg
ala
tyr
cys-cys
val
met
nva
ile
phe
lys
leu
min
262nm,4nm
sar
pro

h

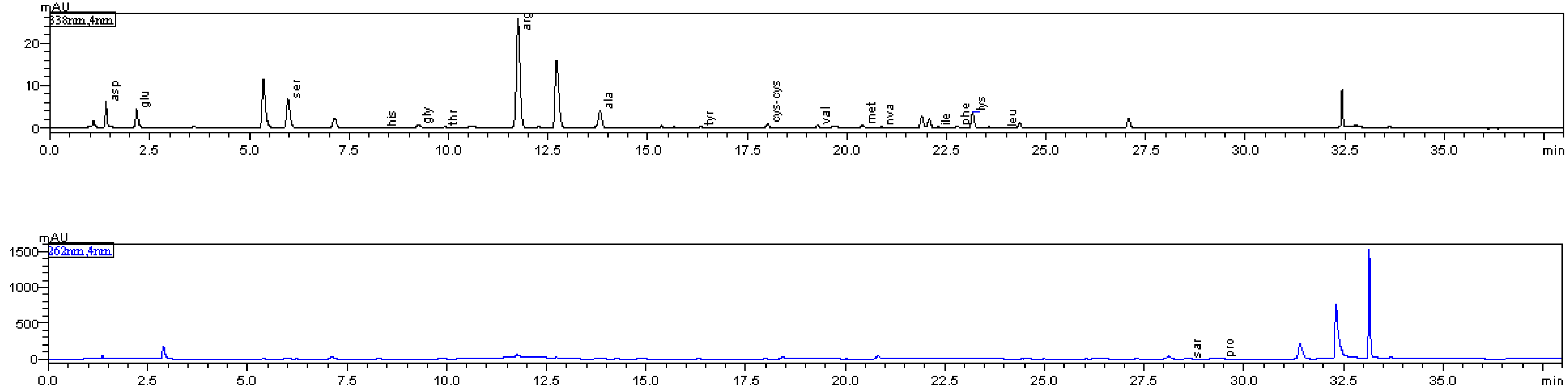

Figure S2. Chromatograms of the amino acid. Standard:Chromatograms of the amino acid standards; A:Chromatograms of the grape berries(RDI-1, 2015)；B:Chromatograms of the grape berries(RDI-2, 2015)；C:Chromatograms of the grape berries(RDI-3, 2015)；D:Chromatograms of the grape berries(CK, 2015)；E:Chromatograms of the grape berries(RDI-1, 2016)；F:Chromatograms of the grape berries(RDI-2, 2016)；G:Chromatograms of the grape berries(RDI-3, 2016)；H:Chromatograms of the grape berries(CK, 2016)； a：Chromatograms of the wines(RDI-1, 2015)；b：Chromatograms of the wines(RDI-2, 2015)；c：Chromatograms of the wines(RDI-3, 2015)；d：Chromatograms of the wines(CK, 2015)；e：Chromatograms of the wines(RDI-1, 2016)；f：Chromatograms of the wines(RDI-2, 2016)；g：Chromatograms of the wines(RDI-3, 2016)；h：Chromatograms of the wines(CK, 2016).

Table S1. Meteorological data and irrigation amount during two years of this study.

	Year	April	May	June	July	August	September	Total
Daily average temperature (°C)	2015	14.54	18.47	23.93	24.8	21.26	17.36	-
	2016	13.81	17.48	24.26	24.50	23.59	15.61	-
ET_0 April 1–September 30 (mm)	2015	119.79	149.96	178.01	171.37	127.92	85.54	832.60
	2016	126.81	143.82	162.44	163.57	141.16	78.47	816.26
Precipitation (mm)	2015	5.8	37.6	22.5	33.5	12.2	32.7	144.3
	2016	25.9	10	48.9	43.4	23.8	34.2	186.2
Solar net radiation(J/cm^2)	2015	2746.13	3448.88	3701.99	3651.97	3128.87	2235.12	-
	2016	2701.32	3318.34	3470.33	3642.65	3305.59	2118.51	-

Note: the meteorological data obtained from micro weather station at experiment site. ET0:calculated from daily meteorological data according to FAO 56 (Allen et al., 1998).

Table S2 Volatile compounds concentration (μg/L) in grape berries from untreated (CK) and treated vineyards.

Compounds	2015				2016			
	RDI-1	RDI-2	RDI-3	CK	RDI-1	RDI-2	RDI-3	CK
Benzaldehyde	3.73±0.1a	3.11±0.04b	3.70±0.09a	2.87±0.06b	1.96±0.13a	1.77±0.12a	1.64±0.11a	2.10±0.14a
Benzyl Alcohol	3.53±0.1ab	2.88±0.04c	3.88±0.06a	3.51±0.13b	2.45±0.14b	4.66±0.27a	3.28±0.19b	3.24±0.19b
Phenylethyl Alcohol	3.44±0.2b	8.7±0.51a	3.41±0.2b	4.68±0.28b	2.91±0.19a	1.49±0.1b	3.71±0.25a	3.55±0.24a
Benzoic acid, 3,5-dimethyl-	2.97±0.18b	1.99±0.12c	3.98±0.23a	1.34±0.08c	3.18±0.19c	3.03±0.18c	6.17±0.36a	4.70±0.28b
Styrene	3.31±0.22b	1.86±0.12c	4.9±0.33a	1.41±0.09c	nd	nd	nd	nd
Hydroquinone	1.2±0.09bc	1.62±0.12b	2.39±0.18a	0.98±0.07c	1.96±0.14b	3.69±0.27a	1.88±0.14bc	1.21±0.09c
**Total amount of aromatic compounds**	18.19±0.07c	20.16±0.32b	22.25±0.03a	14.78±0.29d	12.47±0.13c	14.64±0.2b	16.68±0.17a	14.8±0.08b
3-Pentanol, 3-methyl-	3.9±0.26b	5.77±0.38b	11.03±0.72a	9.45±0.62a	2.17±0.14c	5.03±0.33b	9.11±0.6a	1.96±0.13c
1-Hexanol, 2-ethyl-	3.53±0.25b	1.88±0.14b	6.98±0.5a	8.02±0.58a	nd	nd	nd	nd
Isooctanol	7.18±0.53b	6.04±0.45b	7.49±0.56b	9.98±0.74a	4.33±0.32b	5.09±0.38ab	4.56±0.34b	6.22±0.46a
**Total amount of branched alcohols**	14.61±0.53b	13.69±0.21b	25.5±0.34a	27.45±0.7a	12.6±0.23c	15.7±0.33b	19.69±0.66a	14.26±0.07b
4-Methoxy-4-methyl-2-pentanol	nd	nd	nd	nd	6.10±0.41a	5.58±0.38a	6.02±0.41a	6.08±0.41a
Cyclopentanone, 2-methyl-	542.14±31.11b	734.17±42.14a	459.28±26.36b	438.97±25.19b	589.48±33.83b	855.82±49.12a	703.39±40.37ab	580.2±33.30b
**Total amount of branched ketones**	542.14±31.11b	734.17±42.14a	459.28±26.36b	438.97±25.19b	589.48±33.83b	855.82±49.12a	703.39±40.37ab	580.2±33.3b

Undecanoic acid, hydroxy-, lactone	nd	nd	nd	nd	3.83±0.27a	2.83±0.2b	1.44±0.1c	nd
phenylmethyl acetate	12.78±0.13b	13.98±0.19a	10.81±0.04c	10.67±0.03c	11.40±0.07c	13.47±0.17a	12.47±0.12b	12.82±0.14b
2-methyl-1-butyl acetate	nd	nd	nd	nd	12.00±0.10c	11.88±0.10c	12.70±0.14b	13.38±0.18a
phenethyle acetate	11.04±0.01b	11.97±0.13a	11.49±0.06ab	11.57±0.01ab	11.30±0.05a	11.85±0.16a	11.39±0.01a	11.43±0.02a
isoamyl octanoate	12.38±0.03b	15.28±0.01a	15.36±0.21a	15.65±0.16a	12.93±0.17b	16.35±0.25a	16.68±0.29a	16.92±0.03a
isoamyl hexanoate	11.25±0.08b	13.35±0.03a	13.03±0.11a	13.96±0.21a	11.58±0.01b	14.95±0.29a	15.38±0.34a	15.72±0.17a
**Total amount of branched esters**	47.45±0.13b	54.58±0.19a	50.69±0.04ab	51.85±0.03ab	73.04±0.3b	71.33±0.13a	70.06±0.12a	70.28±0.04a

Different letters in the row indicate significant differences ($P < 0.05$) among treatments for the same vintage

nd: not detected.

Table S3. concentrations of the amino acids of the standard.

Amino acid.	WM	Concentration （μmol/mL）	Concentratio（μg/mL）	Concentration after dilute 5 time （μg/mL）
Asp	133.1	2.5	332.8	66.55
Glu	147.13	2.5	367.8	73.57
Ser	105.09	2.5	262.7	52.55
Gly	75.07	2.5	187.7	37.54
His	155.16	2.5	387.9	77.58
Arg	174.2	2.5	435.5	87.10
Thr	119.12	2.5	297.8	59.56
Ala	89.09	2.5	222.7	44.55
Pro	115.13	2.5	287.8	57.57
Tyr	181.19	2.5	453.0	90.60
Val	117.15	2.5	292.9	58.58
Met	149.21	2.5	373.0	74.61
Cys-Cys	240.3	1.25	300.4	60.08
Ile	131.17	2.5	327.9	65.59
Leu	131.17	2.5	327.9	65.59
Phe	165.19	2.5	413.0	82.60
Lys	146.19	2.5	365.5	73.10
